# Study on co-seismic ionospheric disturbance of Alaska earthquake on July 29, 2021 based on GPS TEC

**DOI:** 10.1038/s41598-023-37374-9

**Published:** 2023-07-01

**Authors:** Qingshan Ruan, Xitun Yuan, Hang Liu, Shuyao Ge

**Affiliations:** 1 Hanzhong Vocational and Technical College, Hanzhong, 723002 China; 2 College of Geomatics, Xi’an University of Science and Technology, Xi’an, 710054 China; 3 School of Geodesy and Geomatics, Wuhan University, Wuhan, 430079 China

**Keywords:** Natural hazards, Seismology, Seismology

## Abstract

With the rapid development of space geodetic information technology, the Global Positioning System (GPS) has been widely used in seismology and space environmental research. Typically, the occurrence of a large earthquake will lead to some changes in the ionosphere, this phenomenon is called coseismic ionospheric disturbances (CIDs). In this contribution, differential slant total electron content (dSTEC) is used to study the anomalous characteristics of the ionosphere. First, based on the ionospheric dSTEC time series and two-dimensional disturbance detection, the temporal and spatial characteristics of ionospheric disturbances can be accurately analysed. Secondly, using wavelet transform spectrum analysis and disturbance propagation velocity, it can be determined that the disturbance sources of this earthquake can be identified as acoustic wave, gravity wave and Rayleigh wave. Finally, in order to further clarify the direction of the earthquake disturbance, this study focuses on proposing an innovative method for the disturbance propagation direction, and determines that there are two directions of the propagation of the CIDs of the Alaski earthquake.

## Introduction

Earthquakes are a natural phenomenon caused by the movement of tectonic plates, resulting in rupture and plate deformation^1^. Owning to the complexity and difficulty of seismic tectonic mechanisms, the study of seismic phenomena and seismic prediction are hotly debated issues. However, with the continued maturation of coupled Global Navigation Satellite System (GNSS) and seismic technology, it is beneficial to detect the characteristics of temporal and spatial perturbations in the ionosphere caused by earthquakes. Currently, a new technology with wide applications in seismology is the GNSS and seismic coupling technology, due to the occurrence of earthquake-induced seismic waves that change the total electron content (TEC) of the ionosphere^2,3^. GNSS technology can be used to invert the TEC before and after the earthquake to improve the accuracy of seismic predictions.

When the ionosphere is unaffected by the external environment, it is in a relatively stable state, and the TEC does not fluctuate, whereas in the ionosphere affected by the external environment, such as earthquakes, magnetic storms^4,5^, volcanoes^6^, and solar flares^7^, anomalies occur. Earthquakes, in particular, are extremely destructive and cause huge losses to society. An earthquake causes a vertical upward seismic wave at the land or ocean surface. When the seismic wave reaches the ionosphere, it changes the total electron content, eventually causing an anomaly in the ionosphere known as a coseismic ionospheric disturbance (CIDs)^8–14^. Currently, scientists have developed a relatively complete theoretical system for studying CIDs. Using GPS technology, Calais and Bernard^15^ observed that ionospheric disturbances near the epicentre of the Northridge earthquake lasted for several minutes and moved away from the epicentre with increasing time from the GPS observation. Further, Heki et al.^16^ found that earthquakes commonly occur in different types of seismic waves and classified three different types of ionospheric anomalies : 1. a sound wave travelling vertically directly from the seismic source; 2. gravity waves caused by earthquakes or tsunamis; 3. a secondary acoustic wave of the Rayleigh wave far from the source area. These conclusions have been widely used and verified by subsequent scholars^17^. For example, Ducic et al.^18^ detected Rayleigh waves in the 2002 Denali earthquake, Astafyeva and Heki^19^ detected two types of seismic waves in the 1994 Kurile earthquake , and Chen et al. discovered two types of seismic waves in the 2015 Nepal earthquake and 2013 Lushan earthquake^20,21^. Liu et al.^22^ used ray tracing technology to detect the abnormal propagation velocity of the ionosphere and the location of the focus of the 1999 Taiwan earthquake. In 2015, Cahyadi and Heki^23^ estimated the velocity of the sound waves from the 2012 Sumatra earthquake to be about 1 km/s. At the same time, other researchers also found that the ionospheric perturbation is asymmetric in the northern and southern hemispheres, i.e. perturbations of northern hemisphere earthquakes occur mainly south of the relative epicentre, and perturbations north of the relative epicentre are not significant^20,24^. Additionally, Kong et al.^25^ used the lithosphere—atmosphere—ionosphere coupling to construct an electric field penetration model to analyse the potential physical mechanism, further confirming the ionospheric anomaly theory. In 2021, Shi et al.^26^ analysed the 2017 Chiapas earthquake, detected an abnormal source location as well as amplitude changes and determined the variation of ionospheric anomalies. Besides, based on GPS TEC technology, relevant scholars have added the new technology of Moderate Resolution Imaging Spectroradiometer (MODIS) Land Surface Temperature (LST) to further detect the changing characteristics of CIDs, and provide new theoretical guidance for earthquake monitoring^27^.

Although GNSS technology has been used to study the Mw8.2 Alaska earthquake, there have been few previous studies on the azimuth of large anomalies in the earthquake, and no specific research on the propagation direction of CIDs^28^. Therefore, based on the study of the disturbance sources and the spatio-temporal variation characteristics of the Alaska earthquake, this paper further quantitatively studies the specific direction of the anomaly propagation of the Alaska earthquake, which provides a certain reference value for the later study of the propagation direction and mechanism of the CIDs of the same earthquake in the high-incidence area, and for the earthquake monitoring. This manuscript presents processed GPS data from 98 Continuously Operating Reference Stations (CORS) in the vicinity of the earthquake, comprehensively analyses the azimuth of the station’s maximum anomaly, determines the specific propagation direction of ionospheric anomalies, and summarises the relationship between the propagation direction and the tectonic plate boundary.

## Data sources and methods

### Earthquake and data overview

**Figure 1:**
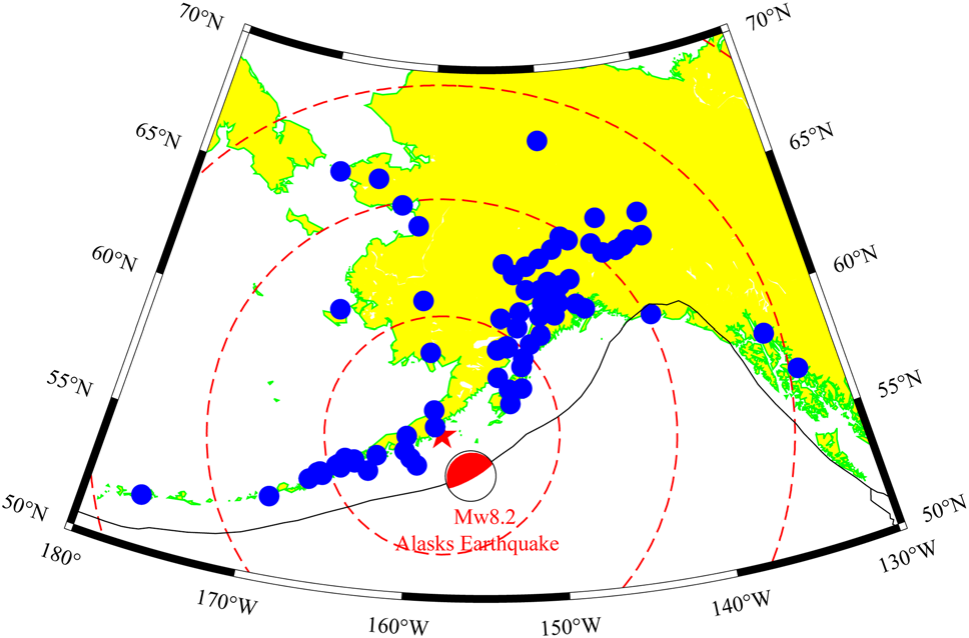
Distribution map of seismic stations in Alaska. ( represents the position of the epicentre,  represents the focal mechanism solution and magnitude,  represents the position of the GPS observing station, and the red dotted line represents the lines of 500 km, 1000 km, 1500 km and 2000 km lines from the epicentre. There are corresponding stations at different distances, and the blue solid line represents the boundary line of the Earth's plate.) The figure was plotted by using the Generic Mapping Tools (vision 5.4.4; URL: https://gmt-china.org/).

On July 29, 2021, at 06:15:41 UT (Universal Time), a Mw8.2 earthquake occurred near the Aleutian Islands, Alaska. The focal depth was about 15 km, and the epicentre was located at 55.40°N, 158.00°W, making it a shallow earthquake accompanied by more than 20 aftershocks. This earthquake is the result of a thrust rupture (about 200 km long and 80 km wide) at near the subduction zone between the Pacific and North American plates. The study comprehensively analyses 98 GPS stations with a 30 s sampling interval provided by the UNAVCO (Fig. 1).

### GPS-TEC processing methods

As mentioned above, the ionosphere is affected by earthquakes, the ionospheric TEC becomes abnormal. In this section, we first calculate the ionospheric TEC based on GPS data from the 98 observing stations at 06:00–07:00 UT. Since the propagation path from the tracking station to each satellite is different, the TEC is different in each direction. Usually, the TEC on the propagation path from the satellite to the tracking station is recorded as the slant total electron content (STEC), and the TEC in the zenith direction is recorded as the vertical total electron content (VTEC), and the VTEC value in the zenith direction is small, and the ionospheric projection function can realise the mutual conversion between STEC and VTEC^29,30^. The height H of the single layer ionospheric modal is assumed to be 350 km. To avoid multipath effects, the satellite cut-off altitude angle is 15°. According to the dual-frequency GPS signal propagation theory, the use of dual-frequency carrier phase observations to calculate the STEC on the signal propagation path is achieved using the following formula^31,32^:

1 $$ STEC = \frac{1}{40.3} \cdot \frac{{f_{1}^{2} f_{2}^{2} }}{{f_{1}^{2} - f_{2}^{2} }}[L_{1} - L_{2} + \lambda_{1} (N_{1} + b_{1} ) - \lambda_{2} (N_{2} + {\text{b}}_{2} ) + \varepsilon_{L} ] $$

where *f*_*1*_ = 1575.42 MHz, *f*_*2*_ = 1227.60 MHz; *L*_*1*_ and* L*_*2*_ are dual frequency carrier phase; $\lambda$_*1*_ and $\lambda$_*2*_ are the GPS carrier signal wavelength; *b*_*1*_* and b*_*2*_ are hardware deviation; *N*_*1*_ and *N*_*2*_ are integer ambiguity; $\varepsilon$ is the residual error.

### Anomaly detection mechanism

When the ionosphere is not disturbed by the external environment, the STEC is relatively smooth when the ground station tracks the satellite, and fluctuates in a short time when the earthquake occurs. Due to the large rate change of STEC, it is difficult to directly detect the CIDs. Therefore, the Gaussian difference STEC method to obtain the relative change of STEC, i.e. dSTEC, is defined as (2)^20^ :

2 $$ \left\{ {\begin{array}{*{20}l} {dSTEC_{i} = STEC_{i + 1} - STEC_{i} } \hfill \\ {dSTEC_{i + 1} = STEC_{i + 2} - STEC_{i + 1} } \hfill \\ \end{array} } \right. $$

where *dSTEC*_*i*_ represents the difference between the STEC at time *i* + 1 and STEC at time *i*, and *dSTEC*_*i*+*1*_ represents the difference between the STEC at time *i* + 2 and STEC at time *i* + 1.

**Figure 2:**
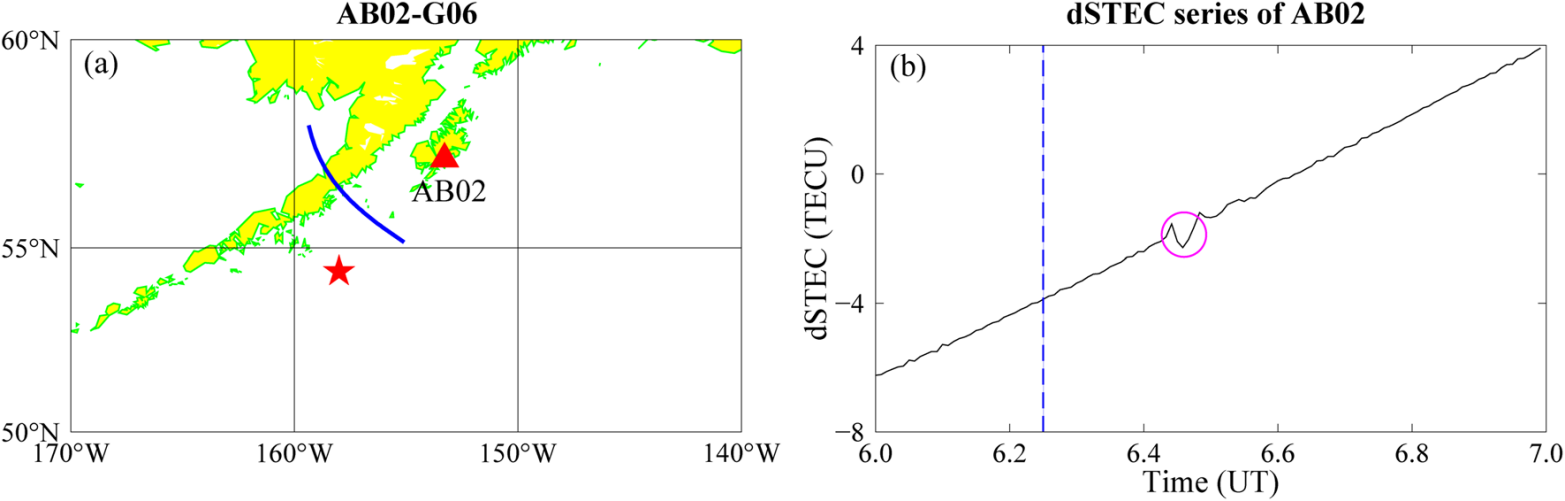
Variation characteristics of the dSTEC time series at 06:00–07:00. (**a**): SIP trajectory of satellite G09 at station AB02. (Red pentagram is the epicentre, red triangle is the AB02 station, blue curve is the SIP trajectory.) (**b**): dSTEC sequence of AB02 station at 06:00–07:00 UT (Blue dotted line is the time of occurrence and magenta circle is the disturbance change). The figure was plotted by using the Generic Mapping Tools (vision 5.4.4; URL: https://gmt-china.org/).

We have chosen satellite G06 at station AB02 in order to further investigate the variability characteristics of the dSTEC time series. Figure 2a shows the location of station AB02 and the sub-ionospheric point (SIP) trajectory of the G06 satellite. Figure 2b shows the dSTEC time series. When the ionosphere is in a stable state, the dSTEC does not show much change. After the earthquake, the seismic waves reach the ionosphere and small fluctuations in the dSTEC appear (as the magenta circles in Fig. 4b), and such small fluctuations are sometimes not easily observed.

**Figure 3:**
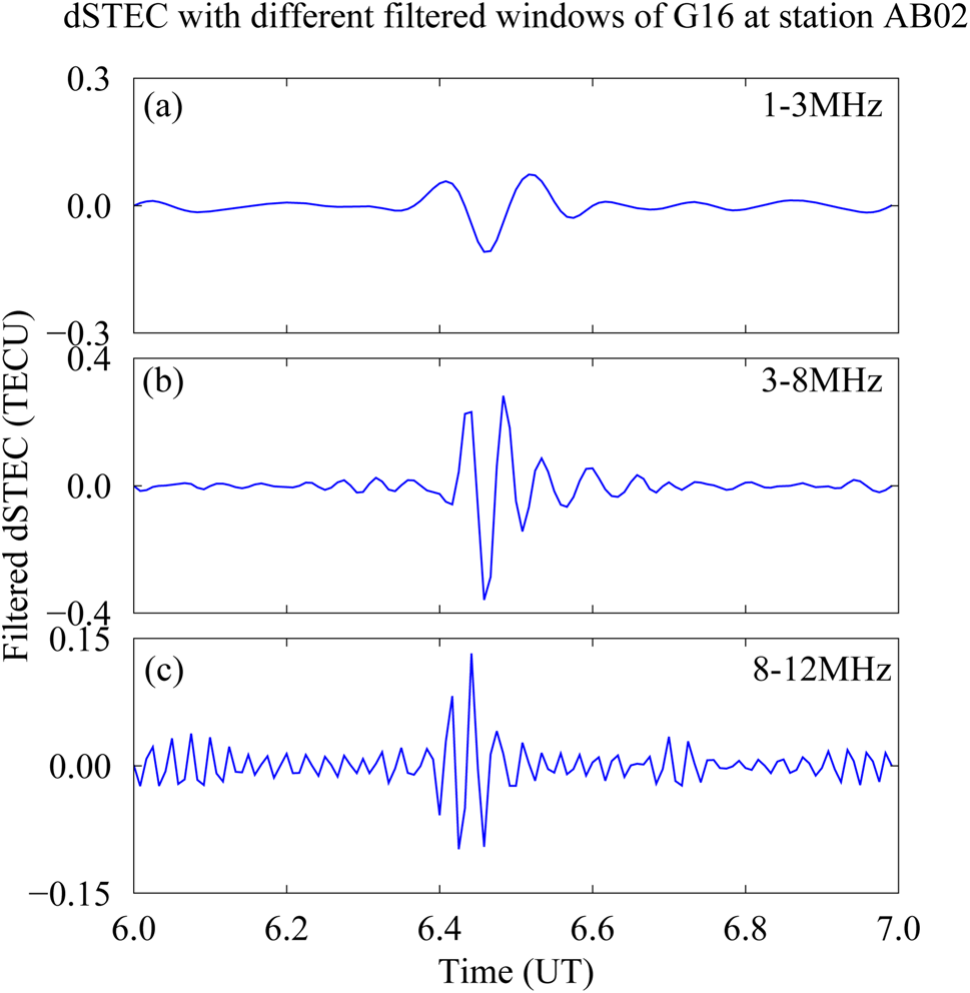
Filter windows for different frequencies. (**a**): 1–3 MHz filter window results. (**b**): 3–8 MHz filter window results. (**c**): 8–12 MHz filter window results.

To analyse the anomalous small changes in the ionosphere, the trend element must be removed from the dSTEC time series. In this study, a band-pass filter is used to allow waves in certain frequency bands to pass through while shielding waves in other frequency bands^19^^.^^33^ . When the dSTEC time series is passed through the wavelet transform band-pass filter, the time series with ionospheric anomalies appear as obvious disturbances. In Fig. 3, the dSTEC time series from satellite G06 at station AB02 is selected for analysis and the 4th order band-pass filter algorithm is applied at 1–3 MHz, 3–8 MHz and 8–12 MHz respectively. The 3–8 MHz filter window is more effective than the 1–3 MHz and 8–12 MHz windows for trend item processing. Thus, this paper selects the 4th order 3–8 MHz band-pass filter to process the change of trend items, which can meet the accuracy of dSTEC after filtering in this paper.

### Solar-geomagnetism index

**Figure 4:**
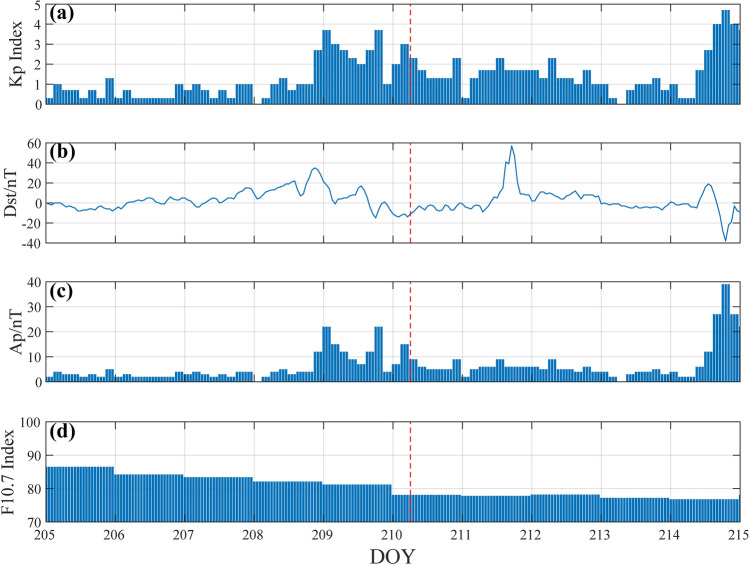
Kp, Dst, Ap, and F10.7 index changes from July 24 to August 3, 2021. The red dotted line represents the time of the Alaska earthquake. (**a**) Kp index change; (**b**) Dst index change; (**c**) Ap index change; (**d**) F10.7 index change.

Before discussing the CIDs of this earthquake, this study considers the influence of factors such as geomagnetic activity and solar activity changes in this area^34^. The Kp index, Dst index, Ap index and F10.7 index are commonly used to study geomagnetic activity and solar activity. The F10.7 index is closely related to solar activity, and the solar activity index F10.7 < 100 SFU in a quiet state. The Dst index reflects the geomagnetic activity in the equatorial region, and the intensity of magnetic storms is divided into strong magnetic storms (Dst < − 100 nT), moderate magnetic storms (− 100 nT < Dst < − 50 nT) and weak magnetic storms (− 50nT < Dst < − 30nT). The Kp index represents the global geomagnetic activity and the Ap index is converted from the Kp index. Figure 4 shows the time series of Kp, Dst, Ap and F10.7 indices within 10 days before and after the earthquake. In the 10 days before and after the Alaska earthquake, the solar activity index F10.7 < 100 SFU, indicating that the solar activity is relatively quiet. Five days before the earthquake, geomagnetic activity was not quiet. Two days ago (28–29 July) there was a weak magnetic storm. The Kp index was about 3.7 nT, the Ap index was about 22 nT, and the Dst index was about 33 nT. It is very likely that a weak magnetic storm occurred. On the day of the earthquake, geomagnetic activity was inactive.

## Discussion

It is often the case that only a small number of satellites pass through the epicentre when seismic waves reach the ionosphere; therefore, only a small number of satellites can detect the ionospheric anomaly. In this study, four satellites (G04, G06, G09, and G16) recorded significant disturbances and were selected to analyse the time series changes of filtered dSTEC after 06:00–07:00 UT. The aim is to obtain the spatial and temporal changes over the Alaska earthquake by analysing the disturbance amplitude, azimuth, and epicentral distance of the maximum anomaly.

### Disturbance time series

To analyse the causes of the CIDs on 29 July, this study constructs time sequence diagrams for different stations from a time perspective. The distance between the station’s maximum anomaly and the epicentral distance is the primary factor, and the epicentral distance of the maximum anomaly for each station from 06:00–07:00 UT is arranged in ascending order. The epicentral distance of each satellite time series gradually increases from bottom to top according to the maximum anomalous value of the station.

**Figure 5:**
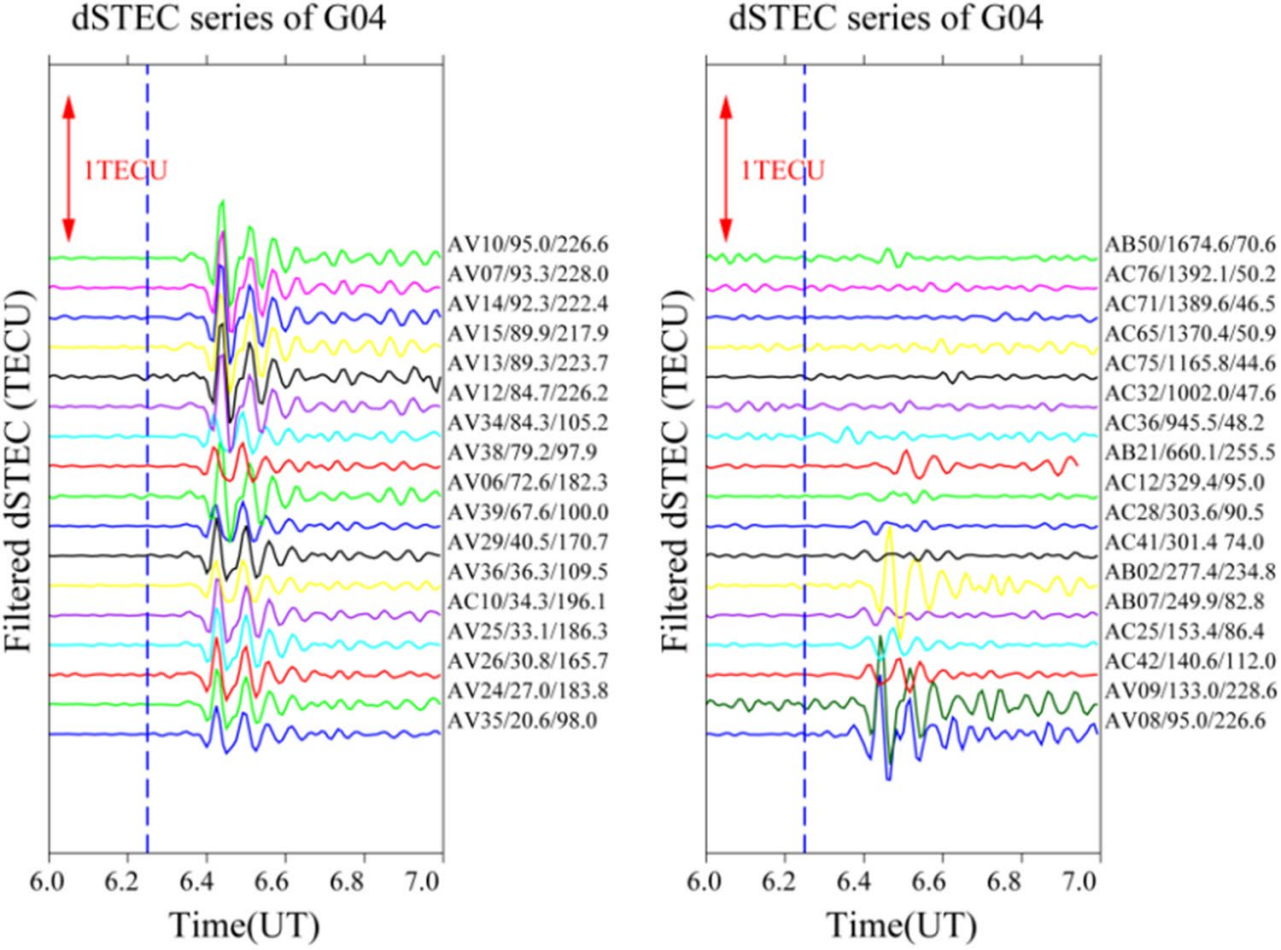
Time series of filtered dSTEC from satellite G04 at 06:00–07:00 UT. (The blue dotted line represents the time of earthquake occurrence, with the name of each station, epicentral distance, and azimuth angle on the right).

Figure 5 shows the filtered dSTEC time series diagram of the satellite G04 at 06:00–07:00 UT. The change in the ionospheric dSTEC before the earthquake is relatively smooth. After the earthquake, the ionosphere fluctuates and gradually returns to normal after a few minutes. The dSTEC changes for most stations after filtering show an anomaly of type “N”, i.e. positive anomaly—negative anomaly—positive anomaly trend. For the stations close to the epicentre, such as AV35, AV24, AV26, AV25, AC10, AV36, and AV29, the perturbation amplitude of the ionospheric anomaly is greater than 0.1 TECU, and the anomaly is mainly concentrated to the southwest and northeast of the epicentre with azimuth angles of 96.0°, 183.6°, 165.7°, 186.3°, 196.1°, 109.5°, and 170.7°, respectively. When the epicentral distance is greater than 300 km, the anomalies are not significant or not visible anomalies (stations AC41, AC28, AC12, AC36, and AC65).

**Figure 6:**
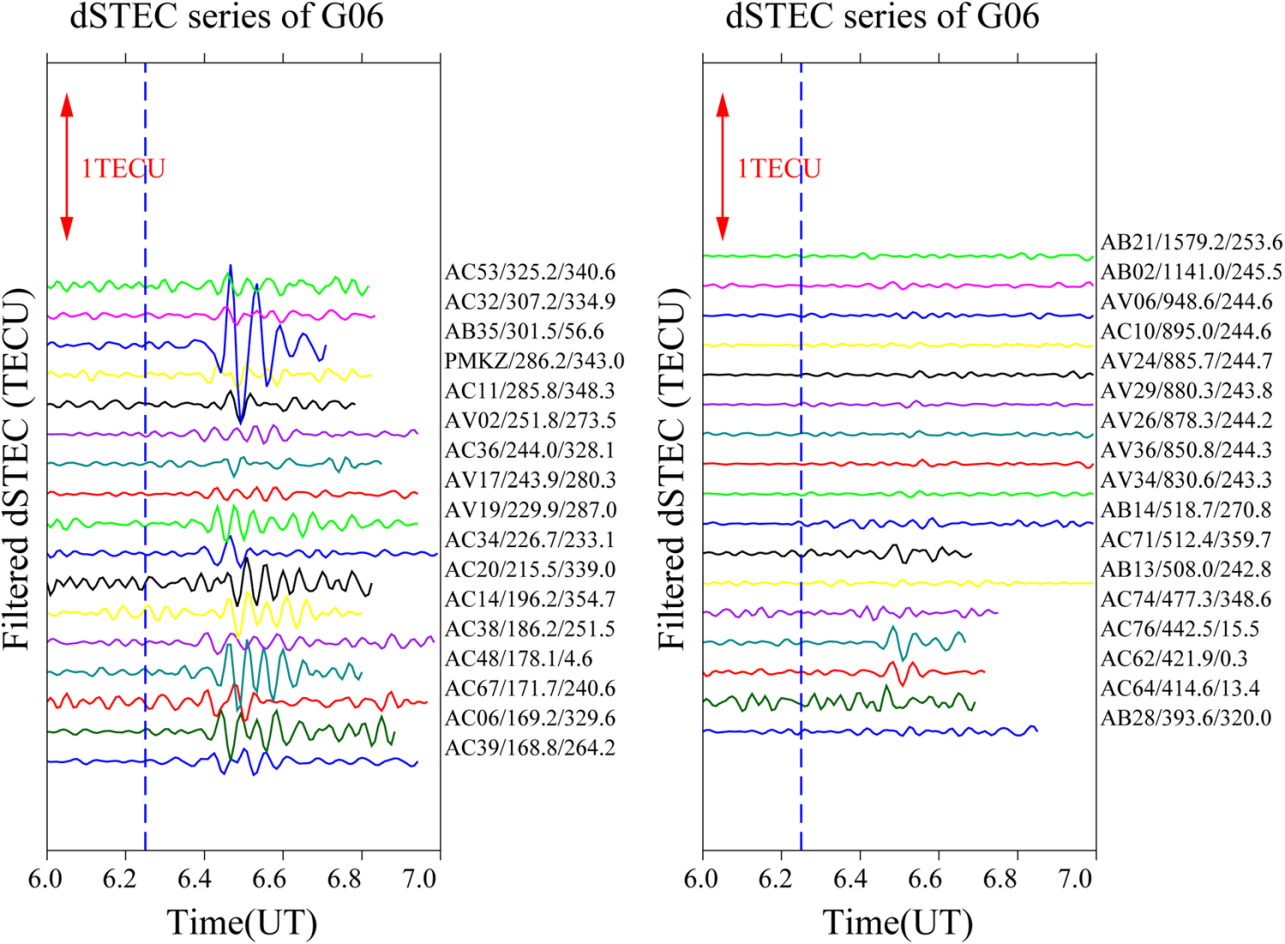
Time series of filtered dSTEC from satellite G06 at 06:00–07:00 UT.

Figure 6 gives the time series for satellite G06 from 34 stations at different distances and azimuths selected for analysis, including stations AC39, AC06, AC67, AC48, AC14, AC20, AC34, and AB35, which are close to the epicentre. The maximum anomaly values varied between 0.0845 and 0.5434 TECU and the anomalies were mainly concentrated the southwest, northwest and northeast of the epicentre with azimuth angles between 240.6° and 56.6°. The ionospheric anomaly of station AB35, located to the northeast of the epicentre (azimuth angle = 56.6°), is particularly significant. The maximum anomaly appears about 13 min after the earthquake and the amplitude reaches 0.5434 TECU, showing that the anomaly intensity of the anomaly varies in different regions in different directions. With increasing epicentral distance (epicentral distance is more than 300 km), weak anomalies appear at stations AC64, AC62, AC76, and AC71. Anomalies often appear to the northeast and north of the epicentre, and other relatively distant stations show no significant or visible anomalies.

**Figure 7:**
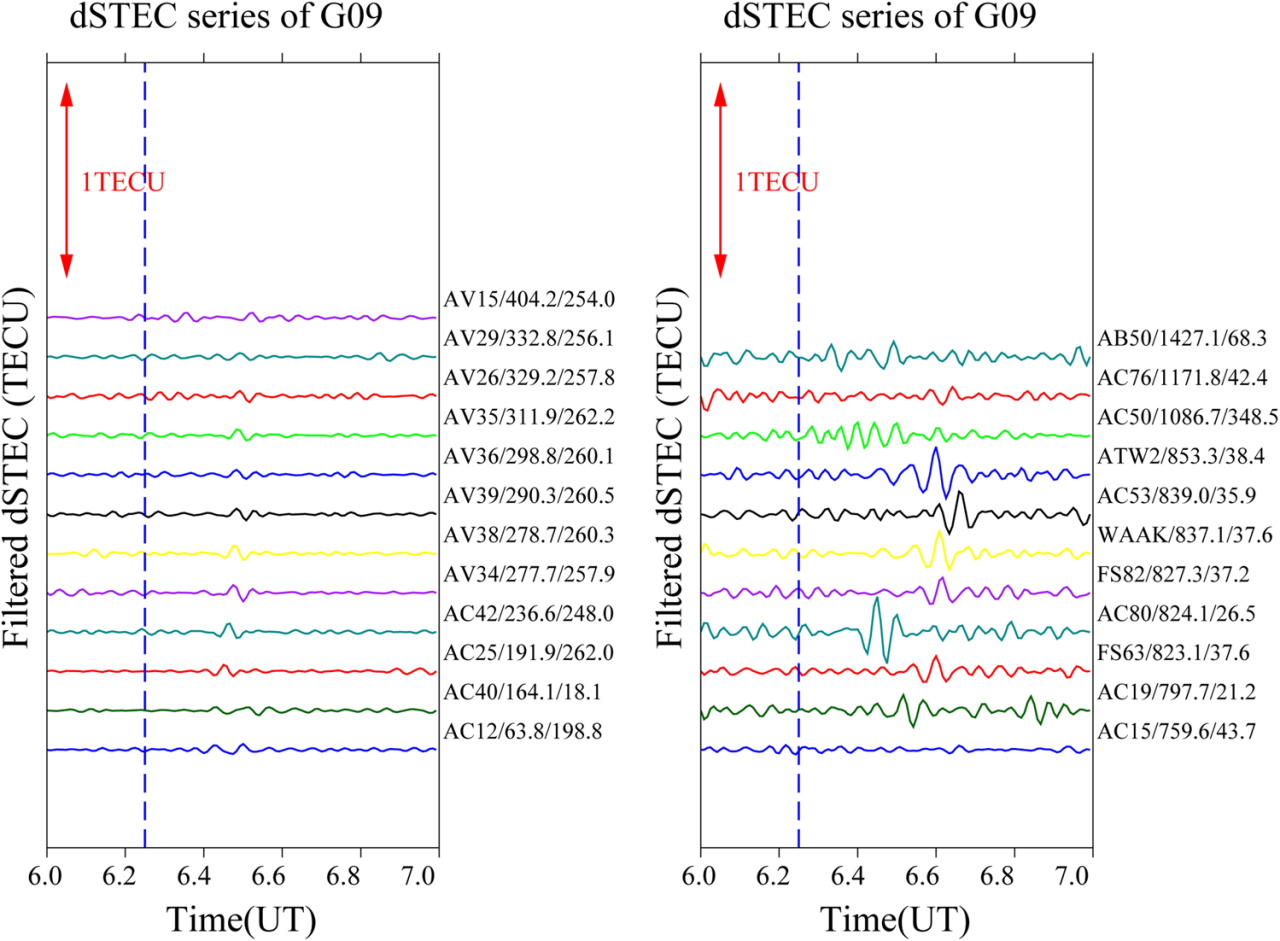
Time series of filtered dSTEC from satellite G09 at 06:00–07:00 UT.

Figure 7 presents the time series for satellite G09. After analysing 23 stations, six stations (AC25, AC42, AV34, AV38, AV39, and AV36) close to the epicentre have weak anomalies to the southwest of the epicentre, showing an “N” type trend with anomalous values of 0.0328, 0.0437, 0.0397, 0.0396, 0.0288, and 0.0247 TECU, respectively. Stations AC19, FS63, AC80, FS82, WAAK, AC53, and ATW2 are located relatively far from the epicentre, but the anomalies are still significant and primarily concentrated to the northeast of the epicentre with azimuth of 21.2°, 37.6°, 26.5°, 37.2°, 37.6°, 35.9°, and 38.4°, respectively. This result is consistent with the anomalous appearance of satellite G06 in the northeast. In addition, the time of occurrence of anomalies at stations FS82, WAAK, AC53, and ATW2 with epicentral distances of 827.3, 824.1, 837.1, and 839.0 km respectively was delayed by about 10 min, and the anomalies were also concentrated to the northeast of the epicentre, which may be related the epicentral distance and azimuth.

**Figure 8:**
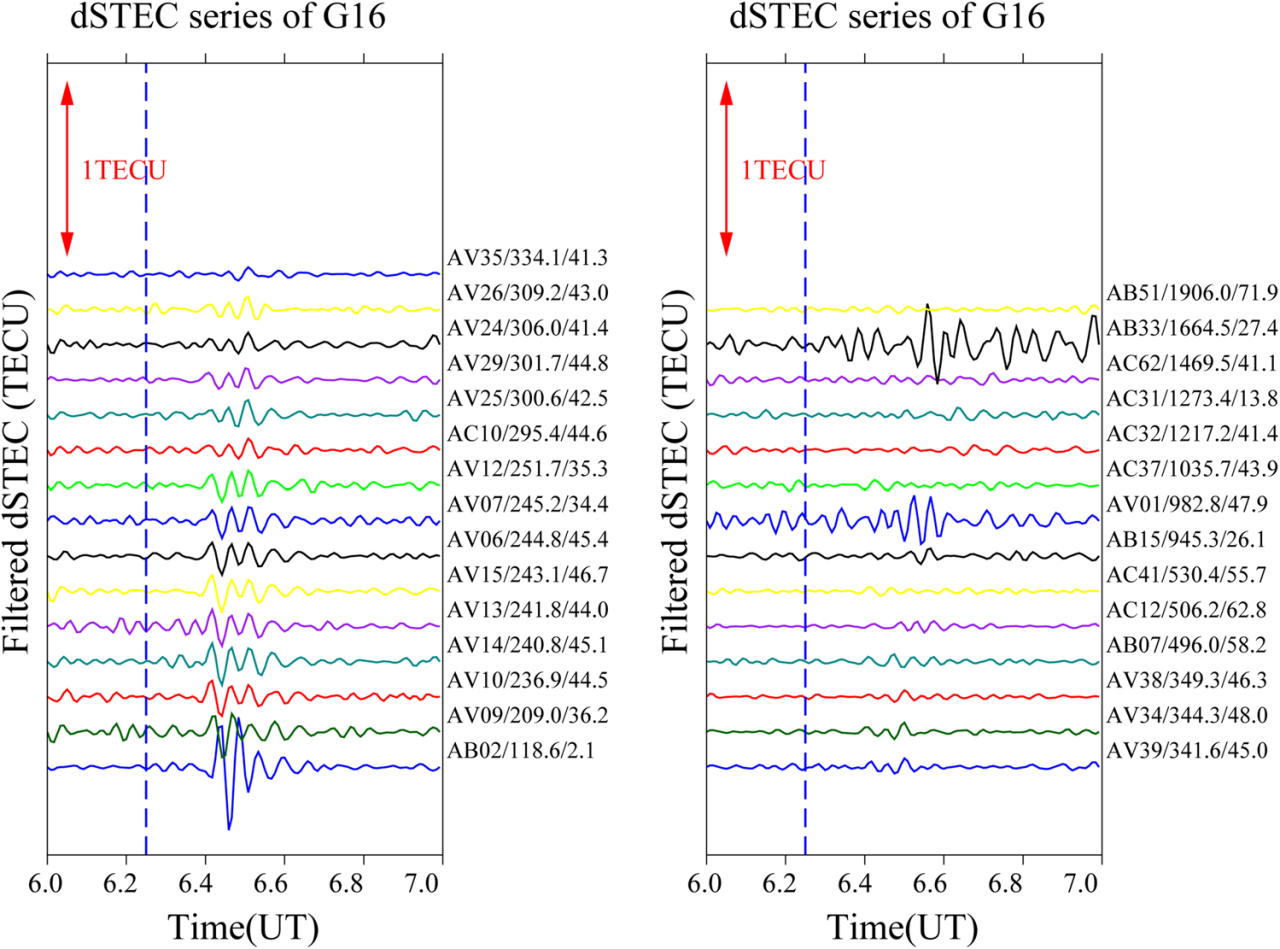
Time series of filtered dSTEC from satellite G16 at 06:00–07:00 UT.

Figure 8 shows the time series for satellite G16. Based on the analysis of 29 stations, it can be seen that there are significant ionospheric anomalies at the stations with an epicentral distance of less than 250 km to the northeast of the epicentre. The anomalies appear 10–13 min after the earthquake with a duration of 5–8 min. The waveform of the anomalies is similar. While stations AV01 and AB33 (with epicentral distances of 982.8 and 1664.5 km, respectively) are far from the epicentre, the anomalies are still concentrated to the northeast of the epicentre (azimuth angles 47.9°, 27.4°), and the anomalies reach 0.1441 and 0.2324 TECU. Other stations recording even weak anomalies are also concentrated northeast of the epicentre, illustrating the close relationship between anomalies and azimuth.

In summary, significant ionospheric “N” type anomalies appear about 10–13 min after the earthquake and are mainly concentrated less than 300 km northeast and southwest of the epicentre. Furthermore, with increasing distance from the epicentre, the disturbance of the maximum number of stations is not significant or even abnormal. Only a few stations show anomalies, and their time of occurrence is delayed by several minutes compared to the stations close to the epicentre. Therefore, it can be speculated that both the epicentral distance and the azimuth angle have a direct influence on the occurrence of anomalies.

### Two-dimensional disturbance analysis

**Figure 9:**
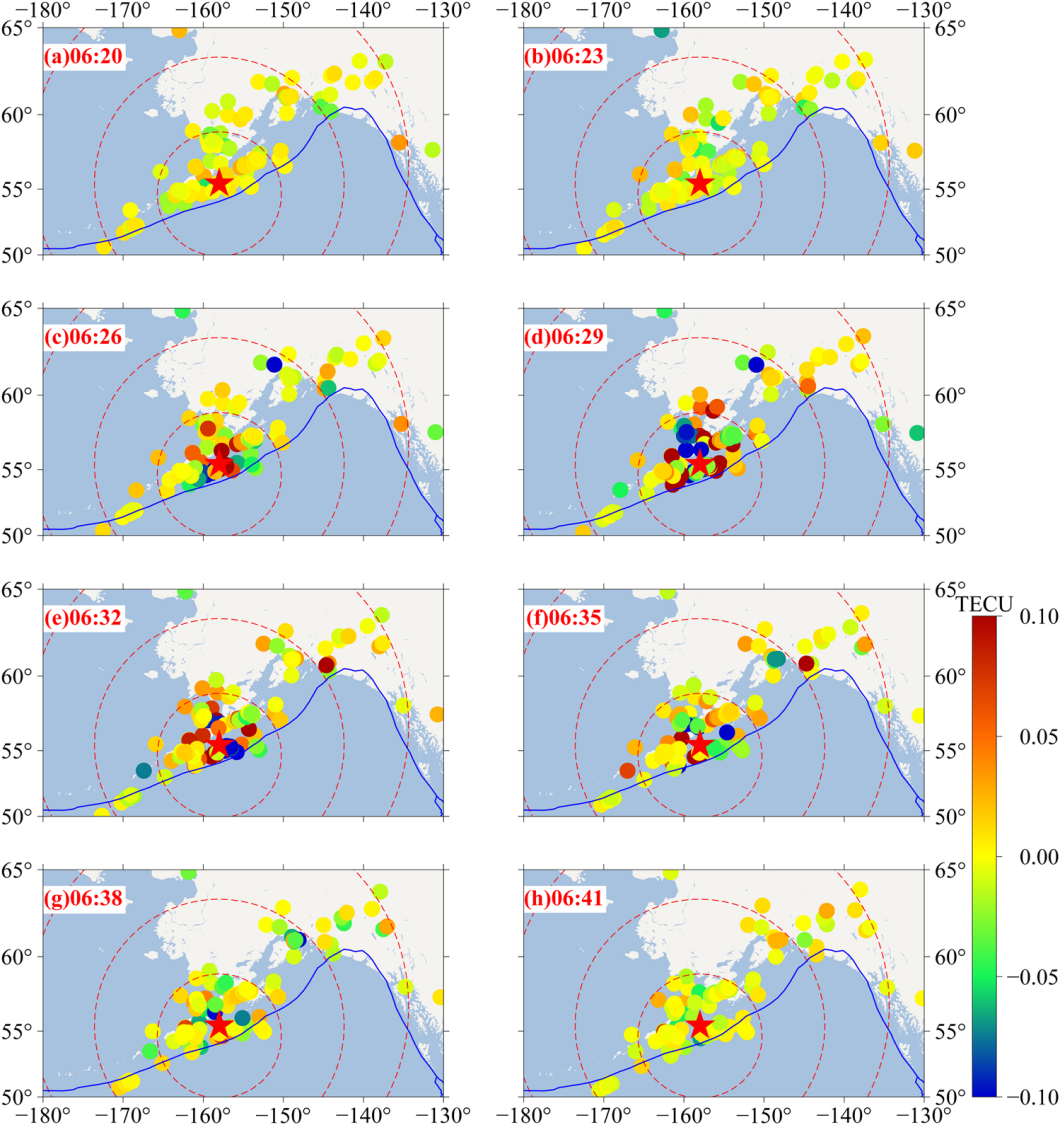
Ionospheric disturbance map over the Alaska earthquake zone. (The blue curve represents the plate boundary; the five-pointed star represents the location of the epicentre; the red dotted line represents the lines at 500 km, 1000 km, 1500 km and 2000 km from the epicentre; the circles of different colours represent the filtered dSTEC values from different stations at a given time.) The figure was plotted by using the Generic Mapping Tools (vision 5.4.4; URL: https://gmt-china.org/).

In order to detect the spatial variation of the CIDs, the two-dimensional ionospheric disturbance at 06:20–06:41 UT on 29 July 2021 over the region after the earthquake is analysed (Fig. 9a–h). The times and trends of the CIDs are markedly different. Within a few minutes after the earthquake, there was no ionospheric disturbance over the region. About 10 min after the earthquake, there was no significant disturbance near the epicentre. At 06:29 UT, there were significant positive and negative anomalies to the northeast and north of the epicentre. During the next three minutes, mainly positive anomalies occurred. At 06:35 UT, the anomaly began to weaken. At 06:38 UT, the ionospheric anomaly disappeared. In total, the anomaly lasted approximately 10 min.

### Disturbance propagation velocity

**Figure 10:**
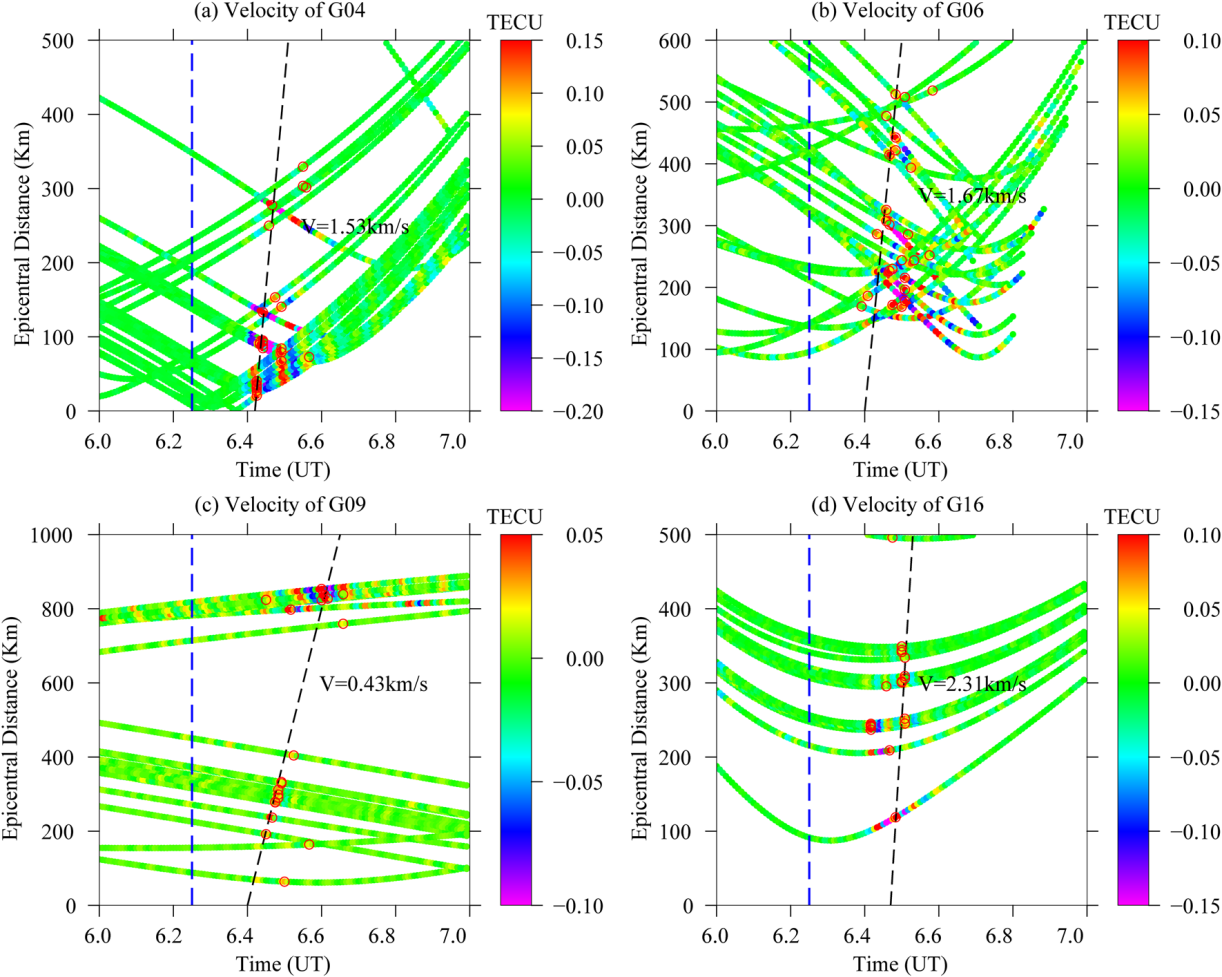
Distribution map of the abnormal propagation velocity of satellites G04, G06, G09 and G16. The red circle represents the position where the maximum abnormal value of a particular satellite appears at 06:00–07:00 UT, the blue dashed line represents the time of the earthquake, and the slope of the black dashed line represents the propagation velocity. The figure was plotted using the Generic Mapping Tools (vision 5.4.4; URL:https://gmt-china.org/). (**a**) Disturbance propagation velocity of satellite G04: 1.53 km/s. (**b**) Disturbance propagation velocity of satellite G06: 1.67 km/s. (**c**) Disturbance propagation velocity of satellite G09: 0.43 km/s. (**d**) Disturbance propagation velocity of satellite G16: 2.31 km/s.

At present, some scientists believe that seismic waves include fast propagation (1.7–2.4 km/s) and slow propagation (0.52–0.68 km/s)^35,36^. According to the seismic wave propagation theory, propagation velocities of 1.53 km/s and 1.67 km/s correspond to the propagation velocity of an acoustic wave (Fig. 10a,b). A propagation velocity of 0.43 km/s corresponds to the velocity of a gravitational wave (Fig. 10c). A propagation velocity of 2.31 km/s, which is greater than that of the acoustic and gravitational waves in Fig. 10a–c, but the propagation velocity is less than the propagation velocity of the Rayleigh wave (about 3.6 km/s). Therefore, the propagation speed of 2.31 km/s is between the two, which may be the result of a mixed superposition wave of acoustic waves and Rayleigh waves. The superposition = $\sqrt{{Aw}^{2}+{Rw}^{2}}$ = $\sqrt{{1.67}^{2}+{3.6}^{2}}$ ≈ 2.8 km/s, which is close to 2.31 km/s in Fig. 10d. Finally, it is concluded that the propagation velocity of 2.31 km/s is a mixed superposition wave formed by the interaction of acoustic waves and Rayleigh waves. Therefore, satellites G04, G06, G09 and G16 all have acoustic waves or gravitational waves, and the satellite G16 has both acoustic waves and Rayleigh waves, but the effect of Rayleigh waves is not clear enough. As can be seen in Fig. 8, the time series of filtered dSTEC from satellite G16 is shown with the corresponding epicentral distance and azimuth for each station on the right. The azimuth of satellite G16 is concentrated around 45°, while the other satellites (G04, G06 and G09) are mainly concentrated around 200°. As the epicentral distances change, the CIDs gradually show a lagging trend. Thus, the propagation velocity of satellite G16, 2.31 km/s, can be directly related to the azimuth relative to the epicentre.

Then, this study analyses the disturbance spectral relationship from the filtered dSTEC time–frequency diagram to illustrate what type of seismic waves caused the CIDs. Stations with different satellite epicentral distances (G04, G06, G09, and G16) are selected for the spectral relationship analysis (Table 1). The time–frequency relationship of the disturbance wavelet transform is then analysed for different stations for different satellites.

**Table 1:** Disturbance of different stations for different satellites.

No.	Station	PRN	Maximum abnormal Time (UT)	Maximum abnormal value (TECU)	Epicentral distance (km)	Azimuth (°)
1	AV15	G04	06:26:30	0.366	89.891	217.9
2	AC75	G04	06:39:30	0.034	1165.763	44.6
3	AC34	G06	06:28:30	0.115	226.676	233.1
4	AC62	G06	06:29:30	0.070	421.862	0.3
5	AV34	G09	06:28:00	0.040	277.730	257.9
6	WAAK	G09	06:34:00	0.113	837.051	37.6
7	AB02	G16	06:29:30	0.283	118.629	2.1
8	AV38	G16	06:30:30	0.037	349.283	46.3

**Figure 11:**
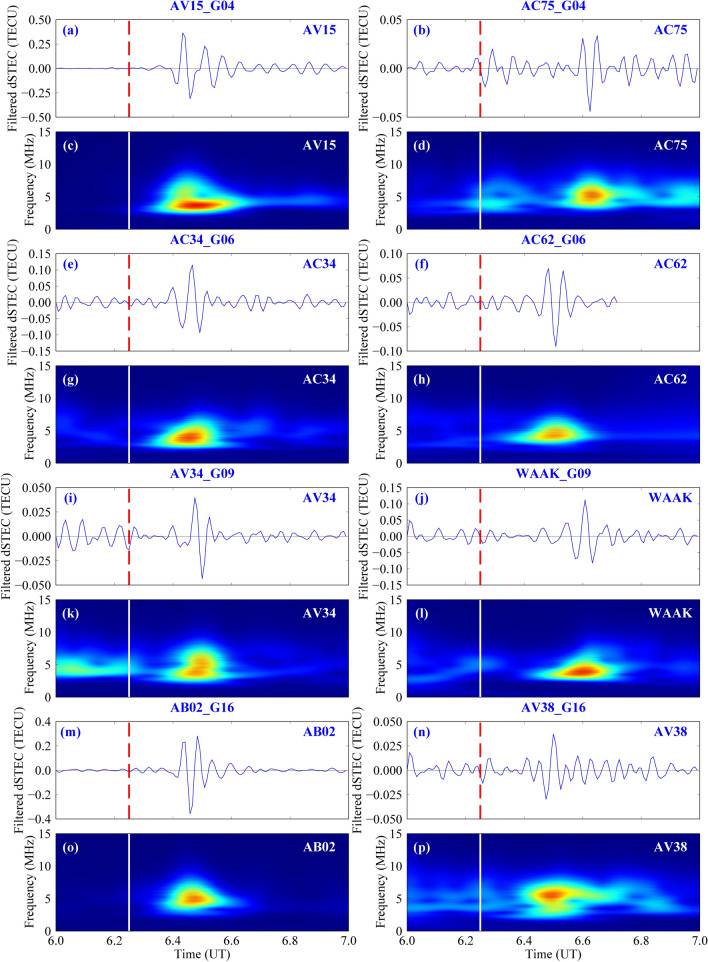
Time–frequency relationship of different stations for satellites G04, G06, G09, and G16. The red dotted line and the white solid line represent the earthquake occurrence time and the frequency plot corresponding to the wavelet transform below the dSTEC time series plot after filtering of each station, respectively. The figure was plotted by using the Generic Mapping Tools (vision 5.4.4; URL: https://gmt-china.org/).

Sequences No. 1 and 2 show a disturbance for stations AV15 and AC75 respectively for satellite G04 (Table 1). The latter has an epicentral distance of more than 1000 km and is mainly located to the northeast of the epicentre (azimuth angle = 44.6°). In comparison, the maximum anomalies of the former are more pronounced (0.366 TECU, 0.034 TECU) and the anomalies are concentrated to the southeast of the epicentre (azimuth angle = 217.9°). Figure 11a–d gives the sequence diagram of dSTEC after filtering at stations the AV15 and AC75 for satellite G04 and the spectrum diagram after wavelet transform. The central frequencies of the anomalous sequences of the two stations are 3.625 MHz and 5.25 MHz respectively (Fig. 11c,d). The former frequency is in the acoustic wave region, while the latter frequency is in the Rayleigh wave region. Therefore, the anomalies of satellite G04 are caused by acoustic waves and Rayleigh waves, further confirming the presence of acoustic waves and Rayleigh waves in the ionospheric anomalies of this earthquake.

Sequences No. 3 and 4 show the disturbance of stations AC34 and AC62 in satellite G06, respectively. Figure 11e,f show that station AC34 is closer to the epicentre than station AC62, with a similar time of the maximum anomaly. The former is located to the southwest of the epicentre (azimuth angle = 233.1°), while the latter is concentrated north of the epicentre (azimuth angle = 0.3°). The centre frequencies of the anomaly sequences from the two stations are 4.125 MHz and 4.4828 MHz respectively, which are in the Rayleigh wave frequency range (Fig. 11g,h).

Sequences No. 5 and 6 show the disturbance of stations AV34 and WAAK for satellite G09. Station AV34 is closer to the epicentre and the time of the maximum anomaly is 6 min earlier than that of station WAAK. The amplitude of the maximum anomaly is smaller than that of station WAAK (the maximum anomaly values are 0.04 TECU and 0.113 TECU respectively). Station AV34 is located southwest of the epicentre and station WAAK is located northeast of the epicentre (Fig. 11i,j). Figure 11k,l show the anomalous spectrums of the two stations. The central frequencies of the anomalous sequences at stations AV34 and WAAK are 5.125 MHz and 4.000 MHz respectively, which is consistent with the Rayleigh wave frequency range.

Sequences No. 7 and 8 show the disturbance of stations AB02 and AV38 for satellite G16. Both stations AB02 and AV38 are located north-east of the epicentre. Station AB02 is closer to the epicentre and the maximum anomaly value is larger. The time of occurrence of anomaly is also 1 min earlier (Fig. 11m,n). The centre frequencies of stations AB02 and AV38 are 5.125 MHz and 5.375 MHz respectively, which are within the Rayleigh wave frequency range (Fig. 11o,p).

The results show that the seismic anomaly is caused by acoustic waves and Rayleigh waves. However, the change in the two-dimensional disturbance can only roughly explain why the large-scale ionospheric anomaly is oriented to the northeast and southwest, and it is difficult to determine the specific propagation direction. The following section focuses on the further determination of the azimuth based on the abnormal azimuth.

### Disturbance azimuth angle

Li et al. studied the propagation direction of the Alaska earthquake ionosphere anomaly and found that the Alaska earthquake anomaly was mainly concentrated in the direction of east-northeast of the epicentre, and the vertical crustal displacement caused by the earthquake rupture was not significant^28^. In order to further detect the ionosphere anomaly of Alaska earthquake, the specific propagation direction of the Alaska earthquake, we apply a new method to study the anomalous propagation direction of the Alaska earthquake. According to the azimuth angle perturbation of the sounding point, if the azimuth angle of a sub-ionospheric point at different stations at different times is used to comprehensively analyse the variation trend of abnormal values, the propagation direction of ionospheric anomalies in this earthquake can be determined. The specific method is to first count the filtered dSTEC with significant anomalies and construct the curve of the filtered dSTEC corresponding to the azimuth angle as well as finding the interval where the abnormal azimuth angle is mainly concentrated from the curve. The mean $\overline{{A{\text{z}}}}$ and variance ${\text{s}}^{2}$ can be used as the approximate disturbance azimuth of the earthquake. The formula is as follows:

3 $$ \overline{Az} = \frac{1}{{\text{n}}}\sum\limits_{i = 1}^{i = n} {Az_{{\text{i}}} } $$

4 $$ {\text{s}}^{2} = \frac{1}{n - 1}\sum\limits_{i = 1}^{n} {(A{\text{z}}_{i} } - \overline{Az} )^{2} $$

where *Az*_*i*_ represents the azimuth angle of all points with significant disturbance at the same time, and the filtered *dSTEC*_*i*_ value is commonly greater than the azimuth angle of 0.05 TECU.

**Figure 12:**
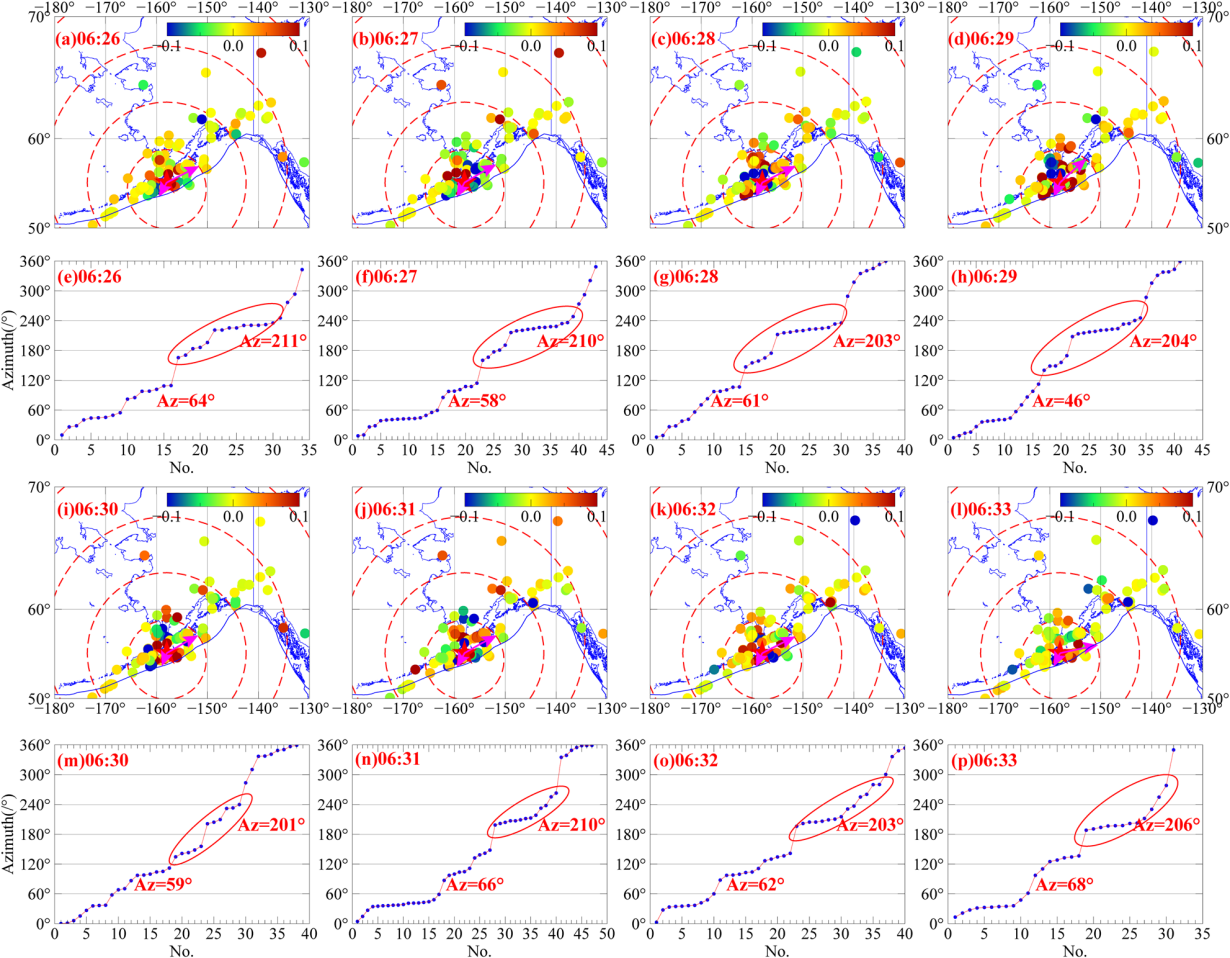
Analysis of the azimuth change of the disturbance in the area from 06:26–06:33 UT. The magenta arrow represents the anomalous direction of the disturbance after fitting, the blue curve is the Pacific Plate margin, and the blue dot represents the maximum anomalous azimuth angle of occurrence. The figure was plotted by using the Generic Mapping Tools (vision 5.4.4; URL: https://gmt-china.org/).

Figure 12 shows the variation of the ionospheric two-dimensional disturbance as well as the variation of the disturbance azimuth from 06:26 to 06:33 UT near the epicentre. Figure 12a–d,i–l,e–h,m–p represent the variations of the ionospheric two-dimensional disturbances and disturbance azimuths from 06:26 to 06:33 UT, respectively. From Fig. 12a–d,i–l, it can be concluded that ionospheric disturbances primarily occur near the epicentre (note: the magenta arrow represents the direction of the fitted disturbance anomaly and the blue curve is the boundary line of the Pacific plate), especially in the period from 06:26 to 06:33 UT, accompanied by positive and negative anomalies. In addition, the line graph fully shows the full azimuth variation of the anomaly (Fig. 12e–h,m–p). The change trends of the 8 graphs are quite similar and the change trend has two parts. One part of the anomaly is mainly concentrated in the red elliptical region, and the other part is mainly concentrated in the azimuth 0°-120°. Thus, the ionospheric anomaly mainly propagates in two directions. However, according to the curves in Fig. 12e–h,m–p, the anomalies are still concentrated to the northeast and southwest of the epicentre. The average azimuth angles at each time point in the corresponding period are 64° and 211°, 58° and 210°, 61° and 203°, 46° and 204°, 59° and 201°, 66° and 210°, 62° and 203°, as well as 68° and 206°, respectively, and there is no significant difference.

In summary, the ionospheric anomalies started about 10 min after the earthquake near the epicentre. At 06:33 UT, the anomalies started to weaken gradually, and the total duration was about 8 min. The anomalies near the epicentre are mainly concentrated to the northeast and southwest of the epicentre and propagate in the directions of 46°-68° and 201°-211°. Since the direction of the anomaly is approximately parallel to the tectonic plate boundary line and the epicentre is close to the Pacific plate boundary line, it is speculated that the direction of the ionospheric anomaly is directly and closely related to the Alaska-Aleutian subduction in the Pacific plate.

## Conclusions

This study uses GPS data from 98 CORS continuous tracking stations to analyse ionospheric anomalies related to the Alaskan earthquake on July 29, 2021. The conclusions are summarized as follows:

1. In terms of temporal variation of dSTEC during the earthquake: significant disturbance appeared 10–13 min after the earthquake near the epicentre, and the duration was 5–8 min. In areas where the epicentre distance is greater than 300 km, a few stations have weak disturbances.
2. Regarding change characteristics of disturbance propagation velocity and wavelet transform spectrum relationship: the seismic propagation velocity after the earthquake reached 0.43–1.67 km/s and was caused by the acoustic wave generated by the earthquake. When the propagation speed is 2.31 km/s, the wave is caused by the mixed superposition of an acoustic wave and Rayleigh wave.
3. For the disturbance propagation direction, the vicinity of the epicentre shows propagation in two directions, namely, to the northeast and southwest of the epicentre after the earthquake. The disturbance azimuth is 46°-68° and 201°-211°, and the azimuth of 46°-68° is approximately parallel to the boundary line of the Pacific plate.

## Data Availability

Publicly available datasets were analysed in this study. The CORS GPS data were provided by the University Navstar Consortium (UNAVCO, ftp://data-out.unavco.org/pub/rinex/obs/), The Kp, Dst, Ap, and F10.7 indexes are available via NASA Goddard Space Flight Center (https://omniweb.gsfc.nasa.gov/form/dx1.html).
